# Positive Supervisor Behaviors and Employee Performance: The Serial Mediation of Workplace Spirituality and Work Engagement

**DOI:** 10.3389/fpsyg.2020.01834

**Published:** 2020-07-24

**Authors:** Alessandro De Carlo, Laura Dal Corso, Francesca Carluccio, Daiana Colledani, Alessandra Falco

**Affiliations:** ^1^Preventive Medicine and Hygiene, Department of Cardiac, Thoracic, Vascular Sciences and Public Health, University of Padua, Padua, Italy; ^2^Department of Philosophy, Sociology, Education and Applied Psychology, University of Padua, Padua, Italy; ^3^Department of Human Science (Communication, Training, Psychology), LUMSA University, Rome, Italy

**Keywords:** positive supervisor behaviors, workplace spirituality, work engagement, employee performance, positive organizations

## Abstract

Organizational research has highlighted the crucial role of supervisors in promoting employee well-being and performance. According to the motivational approach, supervisors positively influence employees’ outcomes by enhancing their positive feelings. In this study, we examine how positive supervisor behaviors may improve employee performance through the serial mediation of workplace spirituality and work engagement. Data were collected from 330 Italian employees. Results showed that supervisor integrity and responsible behaviors have a positive effect on employee performance directly; moreover, positive supervisor behaviors influence performance indirectly, through both the partial mediation of work engagement and the serial mediation of workplace spirituality and work engagement. The present study highlighted that supervisors should behave responsively and honestly to trigger a virtuous motivational process in their employees, which leads to boost their performance. The practical implications of these findings are discussed.

## Introduction

Over the past few decades, supervisor behaviors have been recognized as a key factor for promoting employee performance (Braun et al., 2013; Barrick et al., 2015; Rana and Javed, 2019; Shin and Hur, 2020) and well-being (Liu et al., 2010; Benevene et al., 2018; He et al., 2019). Within the field of positive psychology (Seligman, 1998; Seligman and Csikszentmihalyi, 2000), several much evidence pointed out the crucial role of positive organizational behaviors, starting from positive leadership styles (Luthans, 2002a, b; Avolio et al., 2004). For instance, the transformational leadership style has been studied extensively: results showed its positive relationships with improved performance and reduced stress (Dvir et al., 2002; Wang et al., 2011; Salem, 2015; Ng, 2017). Moreover, a growing body of evidence showed positive relationships between the authentic leadership style and several positive organizational outcomes, such as commitment, job satisfaction, creativity, innovativeness, and performance (Khan, 2010; Azanza et al., 2013; Müceldili et al., 2013; Wong and Laschinger, 2013; Wang et al., 2014; Baek et al., 2019). An authentic leadership style is a positive approach to organizational leadership, characterized by self-awareness, relational transparency, authentic behaviors, and positive moral perspective (Avolio and Gardner, 2005; Walumbwa et al., 2008). Authentic leaders are confident, hopeful, optimistic, resilient, genuine, reliable, motivated by personal convictions, moral/ethical, future-oriented, and aware of their own and others’ values (Gardner et al., 2011). Much research suggests that authentic leaders encourage employees’ development, reduce burnout risk, promote a positive ethical climate, and improve work engagement (Hassan and Ahmed, 2011; Laschinger and Fida, 2014). Moreover, some evidence showed the positive effect of supervisor integrity and authentic leadership on performance (Leroy et al., 2012; Tak et al., 2019). Integrity, emotion management, respect, and a responsible and considerate approach are crucial competencies to manage employees positively and to become positive supervisors (Yarker et al., 2008; Dal Corso et al., 2019b). These competencies are part of a behavioral framework designed to help supervisors in both reducing stress and promoting organizational well-being. This framework revolves around a set of positive macro competencies, given the need for an approach that defines in a practical way the skills to be developed and improved by supervisors. The focus on behaviors has several strengths: for example, behaviors are observable, learnable, and changeable quite easily; it also guides HR management in developing effective interventions. The framework is mainly linked to the transformational, ethical, and authentic models of leadership (Donaldson-Feilder et al., 2011), because the difference between supervisors and leaders is subtle: only supervisors hold a responsibility toward both tasks and other people’s work; the direct link to this strategic responsibility is the essence of the managerial dimension given that leadership does not necessarily entail this concept (Drucker, 2008; De Carlo et al., 2016a).

The literature suggests that several variables are crucial in influencing the relationship between supervisor behaviors and employee performance. Among the main core mechanisms identified in the literature (Ng, 2017), the motivational one is particularly interesting for the present study. According to it, supervisors can stimulate employees to improve their performance by enhancing their feelings of vigor, competence, absorption, and dedication to work. Therefore, this approach suggests that the relationship between positive supervisor behaviors and employee performance may be mediated by positive work feelings, such as the fulfillment of one’s needs or work engagement (Schaufeli and Bakker, 2004; Salanova et al., 2011; Kovjanic et al., 2013).

Over the last few years, workplace spirituality has gained increasing attention in the organizational research field. The literature conceived workplace spirituality as a multidimensional construct and a positive means to improve employees’ well-being (McKee et al., 2011; Kinjerski, 2013). It has beneficial effects across various kinds of organizations, currently influenced by technological innovations (De Carlo et al., 2020) and through environment sharing (Ivaldi and Scaratti, 2019). Spiritual workplaces encourage employees’ sense of community, recognize their spiritual-mystical needs, foster feelings of engaging in meaningful work, and support integrity, respect, responsibility, and personal growth (see Garcia-Zamor, 2003; Giacalone and Jurkiewicz, 2003; Duchon and Plowman, 2005; Badrinarayanan and Madhavaram, 2008; Kinjerski, 2013). Empirical support was found for workplace spirituality as a predictor of several positive organizational outcomes (van der Walt and de Klerk, 2014), such as performance (De Carlo et al., 2016b; Do, 2018; Rahman et al., 2019). Workplace spirituality was found to be positively associated with work engagement, as well (Saks, 2011; Roof, 2015; Gupta and Mikkilineni, 2018; Van Wingerden and Van der Stoep, 2018). Both workplace spirituality and work engagement refer to a sense of entirety and completeness: workplace spirituality posits that employees express their whole inner self at work (Milliman et al., 2003; Duchon and Plowman, 2005), work engagement requests the simultaneous investment of the physical, cognitive, and emotional self (Kahn, 1990). Despite the similarities, much evidence found workplace spirituality to be an antecedent of work engagement (Kolodinsky et al., 2008; Ahmad and Omar, 2015; Petchsawang and McLean, 2017; Hunsaker, 2018; van der Walt, 2018; Hua et al., 2019; Lizano et al., 2019; Baker and Lee, 2020). The more organizations are oriented toward the fulfillment of spiritual needs, the more easily they can engage employees in work (Jurkiewicz and Giacalone, 2004). In particular, Saks (2011) stated that the connection between the two constructs is due to workplace spirituality ability to create the psychological conditions that, according to Kahn (1990), are needed to increase work engagement—namely, psychological meaningfulness, psychological safety, and psychological availability. Bickerton et al. (2015) analyzed the connection between workplace spirituality and work engagement in the JD-R model and found that spiritual resources had a significant motivational effect: mystical experience at work increased employees’ work engagement and reduced their intention to quit the organization.

Work engagement is a positive state of vigor, dedication, and absorption of employees with their work (Bakker and Demerouti, 2008). Engaged employees have a sense of energetic and effective connection with their activities, feel competent and effective on their job (Schaufeli et al., 2006), and experience a fulfilling state of mind (Bakker et al., 2008). Work engagement has been largely studied in the framework of positive organizational psychology. Positive associations were found with several positive outcomes, such as improved performance (e.g., Christian et al., 2011; Bakker et al., 2012; Reijseger et al., 2017), greater organizational commitment (e.g., Hakanen et al., 2008; Saks, 2011; Qodariah Akbar and Mauluddin, 2019), increased levels of well-being (e.g., Schaufeli et al., 2008; Shimazu and Schaufeli, 2009; Joo and Lee, 2017), and reduced intention to quit (e.g., Schaufeli and Bakker, 2004; Li et al., 2019). Much research, in addition, devoted efforts to identify antecedents of work engagement. Results highlighted the positive effect of job resources, such as autonomy, value congruence, social support from colleagues and supervisors, trusting relationships with supervisors, and workplace spirituality (Bakker and Demerouti, 2008; Rich et al., 2010; Saks, 2011; Falco et al., 2013; Barbieri et al., 2014; Roof, 2015; Gill et al., 2019).

This study aims to analyze how positive supervisor behaviors are linked to employee performance. Specifically, we aim to examine the serial mediation of workplace spirituality and work engagement in the relationship between positive supervisor behaviors and employee performance.

## Materials and Methods

### Participants and Procedure

A total of 330 completed questionnaires were collected (males = 237; mean age = 39.61, SD = 9.06) from five different Italian companies (23.9% banking; 1.8% large-scale retailing; 32.1% oil and gas; 15.5% chemical, industrial, and pharmaceutical; 26.7% metalworking). The majority of participants were white collars (78.5%; blue collars 20%; missing 1.5%) with a seniority in their company lower than ten years (58.5%; over 10 years 39.7%; missing 1.8%). A questionnaire consisting of three standardized scales was administered to measure positive supervisor behaviors, workplace spirituality, and work engagement. The questionnaires were administered on-site and they were filled out in pencil by the participants. A researcher, present in the room during the administration, collected the questionnaires. Participants were asked to rate their performance. Our study was conducted following the recommendations of the Ethics Committee of Psychology Research of the University of Padua. All participants were duly informed that participation was anonymous and voluntary.

### Measures

Positive supervisor behaviors were assessed through the first scale of the Stress Management Competency Indicator Tool (SMCIT; Donaldson-Feilder et al., 2009). The scale includes 17 items scored on a five-point scale (from “strongly disagree” to “strongly agree”) and evaluates three dimensions: integrity (e.g., “This Manager is honest”), emotions management (e.g., “This Manager doesn’t pass on their stress to the team”), and considerate approach (e.g., “The deadlines this Manager creates are realistic”). High scores in this scale describe respectful and honest supervisors with clear values. These supervisors support employees, manage them thoughtfully, and behave consistently and calmly, as good role models. The Cronbach’s alpha for the scale is 0.89.

Workplace spirituality was evaluated through the Spirit at Work Scale (SAWS; Kinjerski, 2013). The instrument includes 18 items scored on a six-point scale (from “completely untrue” to “completely true”). The SAWS evaluates the experience of workplace spirituality through four subscales: engaging work (e.g., “I am passionate about my work”), sense of community (e.g., “I feel like I am part of ‘a community’ at work”), spiritual connection (e.g., “My spiritual beliefs play an important role in everyday decisions that I make at work”), and mystical experience (e.g., “I experience moments at work where everything is blissful”). The Cronbach’s alpha for the scale is 0.91.

Work engagement was assessed by the shortened Italian version of the Utrecht Work Engagement Scale (UWES-9; Balducci et al., 2010; see also Schaufeli et al., 2006). The instrument comprises three subscales, with three items each: vigor (e.g., item “At my job, I feel strong and vigorous”), dedication (e.g., item “My job inspires me”), and absorption (e.g., item “I feel happy when I am working intensely”). The items were rated on a six-point scale (from “never” to “always”). The Cronbach’s alpha for the scale is 0.91.

Employee performance was evaluated through two self-report items. Specifically, participants were asked to evaluate their performance on a 10-point scale (from “low” to “high”) and to rate the work objectives achieved in the last year through a percentage (from 0 to 100%). The Cronbach’s alpha for the scale is 0.69.

### Statistical Analyses

We examined the relationships between positive supervisor behaviors, workplace spirituality, work engagement, and employee performance through structural equation modeling. In the tested model, positive supervisor behaviors were the predictor, workplace spirituality was the first-order mediator, work engagement was the second-order mediator, and employee performance was the criterion variable. Three to four parcels were computed to define the constructs (parcels were created by averaging items of the subscales of the different constructs), while a two-item indicator was employed to measure employee performance. The analyses were run using the Mplus7 package (Muthén and Muthén, 2012) and the maximum likelihood as an estimator. In the mediation model, all paths were estimated and the 95% bootstrap confidence interval (5000 bootstrap samples) was used to test the significance of the indirect effect.

To evaluate the model, several goodness-of-fit indices were used: χ^2^, comparative fit index (CFI; Bentler, 1990), standardized root-mean-square residual (SRMR; Bentler, 1995), and root-mean-square error of approximation (RMSEA; Browne and Cudeck, 1993). Concerning χ^2^, a solution fits the data well when the value is non-significant (*p* ≥ 0.05). Because this statistic is sensitive to the sample size, inspection of the other fit indices is recommended. In particular, a good fit is supported by CFI indices close to 0.95 (0.90–0.95 for a reasonable fit), SRMR values less than 0.08, and RMSEA smaller than 0.06 (0.06–0.08 for a reasonable fit; Hu and Bentler, 1999; Marsh et al., 2004; Brown, 2006).

## Results

Descriptive statistics and reliability indices of all scales are reported in Table 1.

**TABLE 1:** N items, mean, *SD*, and alpha coefficients for all scales used.

	***N items***	**Mean**	***SD***	**Alpha**
Integrity	5	3.87	0.62	0.84
Managing emotions	6	3.52	0.62	0.81
Considerate approach	6	3.49	0.52	0.64
**Positive supervisor behaviors**	**17**	**3.63**	**0.51**	**0.89**
Engaging work	7	4.31	0.77	0.85
Sense of community	3	4.47	0.78	0.75
Spiritual connection	3	3.69	1.03	0.74
Mystical experience	5	3.97	0.76	0.71
**Workplace spirituality**	**18**	**4.14**	**0.69**	**0.91**
Vigor	3	4.23	0.86	0.81
Dedication	3	4.44	0.87	0.89
Absorption	3	4.48	0.75	0.80
**Work engagement**	**9**	**4.38**	**0.73**	**0.91**
Self-rated performance	2	88.54	12.48	0.69^†^

*^†^Concerning self-rated performance reliability was computed through Spearman–Brown coefficient. Normal values indicate sub-scale scores. Bold values show the total scores of positive supervisor behaviors, workplace spirituality, and work engagement.*

**FIGURE 1:**
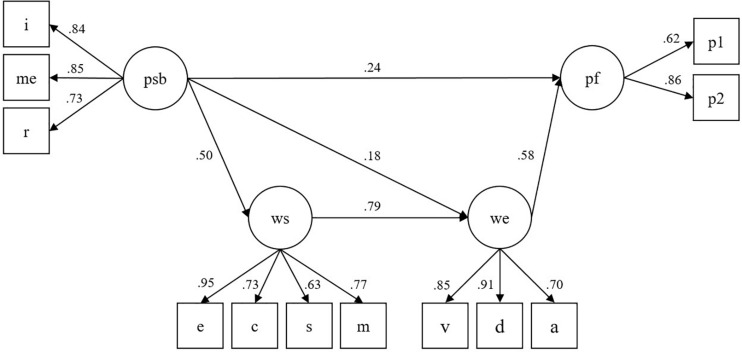
The model tested. *Note.* Psb, positive supervisor behaviors (parcels: i = integrity, me = managing emotions, r = considerate approach); ws, workplace spirituality (parcels: e = engaging work, c = sense of community, s = spiritual connection, m = mystical experience); we, work engagement (parcels: v = vigor; d = dedication; a = absorption); pf, employee performance (p1, p2 = items-indicator). Standardized coefficients; all values are significant *p* ≤ 0.01 (only significant paths are represented).

The model tested is represented in Figure 1 and fits the data well: χ^2^(48) = 94.684, *p* ≅ 0.000; RMSEA = 0.054, CFI = 0.979; SRMR = 0.032. Results show a direct positive effect of positive supervisor behaviors on employee performance (95% CI = 0.076, 0.421) and two indirect effects mediated by workplace spirituality and work engagement (Table 2). Specifically, positive supervisor behaviors have a positive effect on work engagement (95% CI = 0.079, 0.275), which partially mediates the effect of positive supervisor behaviors on employee performance (95% CI = 0.027, 0.201). In addition, positive supervisor behaviors have a positive effect on workplace spirituality (95% CI = 0.389, 0.590), which partially mediates the effect of positive supervisor behaviors on work engagement (95% CI = 0.299, 0.483). Positive supervisor behaviors, therefore, have positive effects on employee performance through the partial mediation of work engagement (95% CI = 0.027, 0.201) and through the serial mediation of both workplace spirituality and work engagement (95% CI = 0.083, 0.406). The relation between workplace spirituality and employee performance is totally mediated by work engagement (95% CI = 0.173, 0.784).

**TABLE 2:** Table of direct and indirect effects.

**Direct effects**	**95% CI**	**Point estimate**
Psb → employee performance	0.076, 0.421	0.244
Psb → workplace spirituality	0.389, 0.590	0.496
Psb → work engagement	0.079, 0.275	0.183
Workplace spirituality → work engagement	0.704, 0.870	0.787
Work engagement → employee performance	0.221, 0.958	0.579
**Indirect effects**		
Psb → work engagement → employee performance	0.027, 0.201	0.106
Psb → workplace spirituality → work engagement → employee performance	0.083, 0.406	0.226
Psb → workplace spirituality → work engagement	0.299, 0.483	0.390
Workplace spirituality → work engagement employee → performance	0.173, 0.784	0.456

*Psb, positive supervisor behaviors. Standardized coefficients; CI 95%, confidence intervals 95%.*

## Discussion

The present study aimed to analyze how positive supervisor behaviors were linked to employee performance. Specifically, we examined the mediation role of workplace spirituality and work engagement in the relationship between positive supervisor behaviors and employee performance.

The literature suggested that positive supervisors positively influenced employees’ outcomes through a motivational process mediated by employees’ positive feelings, such as the fulfillment of one’s needs and work engagement (Schaufeli and Bakker, 2004; Salanova et al., 2011; Kovjanic et al., 2013; Ng, 2017; Dal Corso et al., 2019a). The results of the present study supported this perspective.

We found that positive supervisor behaviors had a direct positive effect on workplace spirituality, work engagement, and performance. Workplace spirituality is a multifaceted construct, which has only recently been introduced in the organizational research field. Research conducted in the last few years showed its positive effects on several positive outcomes, such as reduced burnout, increased well-being, and job satisfaction (McKee et al., 2011; Kinjerski, 2013; van der Walt and de Klerk, 2014). In addition, several studies observed a positive effect of workplace spirituality on employee performance (Garcia-Zamor, 2003; Giacalone and Jurkiewicz, 2003). In the present study, workplace spirituality partially mediated the relationship between positive supervisor behaviors and work engagement, which, in turn, totally mediated the relationship between workplace spirituality and performance, confirming the crucial role of work engagement in promoting positive outcomes. Indeed, work engagement is a positive work-related motivational state associated with other favorable outcomes, such as organizational commitment, employee well-being, and reduced stress (Saks, 2006; Hakanen et al., 2008; Schaufeli et al., 2008; Shimazu and Schaufeli, 2009).

Moreover, our results showed that positive supervisor behaviors had positive indirect effects on employee performance through both the partial mediation of work engagement and the serial mediation of workplace spirituality and work engagement, respectively. In particular, supervisor integrity and ability in managing emotions was found to be crucial to increase work engagement, thus leading to enhanced employee performance. At the same time, workplace spirituality contributed to explain the relationship between positive supervisor behaviors and work engagement. Because work engagement mediated the relationship between positive supervisor behaviors and employee performance, and workplace spirituality mediated the relationship between positive supervisor behaviors and work engagement, the serial mediation is confirmed. Therefore, workplace spirituality and work engagement are important links in the chain that contributes to explain the relationship between positive supervisor behaviors and employee performance.

Although our results are interesting, some limitations can be recognized, for example, the cross-sectional nature of the study. Future studies should try to extend our findings using a longitudinal design. Furthermore, studies with wider and different samples could be useful to circumscribe the possible effects of the specific sample of this study. Indeed, our sample of mainly white collars could have increased the effect of workplace spirituality and work engagement because of their education level and the inherent nature of their job. Besides that, the fact that the participants were completely from Italian companies can make the spirituality effect influenced by the Italian history and culture. Finally, our sample of female workers is still smaller than males, and that should be expanded in the future. In addition, future research should attempt to replicate our results using not only self-report measures but also other data sources (Falco et al., 2012, 2018). Moreover, future studies are needed to understand how positive supervisor behaviors may be trained to foster workplace spirituality and, consequently, its positive outcomes. Finally, future research could investigate the association between positive supervisor behaviors and workplace spirituality on the one hand and negative forms of heavy work investment (e.g., workaholism; Falco et al., 2017) on the other.

## Practical Implications

The findings of the present study suggest that supervisors have a great responsibility toward employees’ well-being because they shape the working environment through their daily behaviors (Girardi et al., 2015). Top management should help supervisors to identify misbehaviors by suggesting better alternative conduct. At the same time, organizations should plan adequate training activities to improve supervisors’ positive competencies (Ivaldi and Scaratti, 2015) and set positive examples on which all supervisors can model their behaviors. The importance of involving the entire organization is decisive in creating a positive organizational culture. The role of organizations is also to emphasize the significance of positive management, specifying that it is not an additional burden to be met, but a set of competencies and attitudes to be valued and integrated into the day-to-day management of employees and tasks. Once this shared awareness has been created, positive supervisors will fulfill employees’ spiritual needs and foster the motivational process that enhances their performance.

## Conclusion

The present findings highlight the importance of positive supervisor behaviors in promoting employee well-being. Moreover, they support the crucial role of work engagement in the relationship between positive supervisor behaviors and employee performance, and provide a new contribution by taking into account the role of workplace spirituality.

## Data Availability Statement

The raw data supporting the conclusions of this article will be made available by the authors, without undue reservation.

## Ethics Statement

Ethical review and approval was not required for the study on human participants in accordance with the local legislation and institutional requirements. The patients/participants provided their written informed consent to participate in this study.

## Author Contributions

AD developed the research project, with the contribution of LD and AF. AD and LD reviewed the literature, with the contribution of AF and FC. FC and DC prepared the dataset and carried out the data analysis. All authors contributed to the article and approved the submitted version.

## Conflict of Interest

The authors declare that the research was conducted in the absence of any commercial or financial relationships that could be construed as a potential conflict of interest.
